# Computational discovery of BRD4 inhibitors for neuroblastoma therapy using pharmacophore screening and molecular simulations

**DOI:** 10.1038/s41598-025-20714-2

**Published:** 2025-10-22

**Authors:** Iqra Ali, Mervt Almostafa, Faheem Abbas, Nancy S. Younis, Azmat Ali Khan, Galal Yahya

**Affiliations:** 1 Department of Biosciences, COMSATS University Islamabad, Islamabad Campus, Islamabad, 45550 Pakistan; 2 Department of Chemistry, College of Science, King Faisal University, 31982 Alhofuf, Al-Ahsa Saudi Arabia; 3 Department of Chemistry, Tsinghua University, Beijing, 100084 China; 4 Department of Pharmaceutical Sciences, College of Clinical Pharmacy, King Faisal University, 31982 Alhofuf, Al-Ahsa Saudi Arabia; 5 Pharmaceutical Biotechnology Laboratory, Department of Pharmaceutical Chemistry, College of Pharmacy, King Saud University, 11451 Riyadh, Saudi Arabia; 6 Department of Microbiology and Immunology, Faculty of Pharmacy, Zagazig University, Al Sharqia, 44519 Egypt; 7 Molecular Biology Institute of Barcelona (IBMB), CSIC, Barcelona, Spain

**Keywords:** Bromodomain-containing protein 4, Pharmacoinformatic approach, Virtual screening, ADMET, Molecular dynamics (MD) simulation, Cancer, Computational biology and bioinformatics, Drug discovery

## Abstract

BRD4 (“Bromodomain-containing protein 4”), a recognized gene regulator, is an attractive target for therapeutic development, particularly for the management of neuroblastoma. An integrated pharmacoinformatic strategy for the development of new BRD4 inhibitors is examined in this research. Pharmacophores were used to digitally screen five databases, and the current study aims to determine the best binding modes by docking the screened hits to the BRD4 active site. Using the BRD4 protein co-crystal ligand (73B) (PDB ID: 4BJX) as a template, pharmacophore hypotheses were produced. Five databases were subjected to a pharmacophore-based virtual screening process, and 1089 hits that satisfied the screening requirements were selected for docking against the BRD4 receptor by using the SP module of the Glide tool. The top ten docked compounds with the highest binding affinities, ranging from − 9.623 to − 8.894 kcal/mol, were selected. Further, the biological activity and ADMET analysis revealed that the selected compounds have values that fall in the acceptable range. The protein-ligand complexes’ stability was verified by performing molecular dynamics (MD) simulations of the binding positions of the top two compounds against the BRD4 receptor. The stability and binding free energies of the compounds indicate that these compounds may function as lead compounds to affect the biological activity of BRD4 in the in vitro studies.

## Introduction

Neuroblastoma is a cancer that arises from immature nerve cells in various parts of the body. It is mostly found in and around the adrenal glands, which are located above the kidneys. This cancer commonly affects children, particularly those under the age of five. The aggressiveness of neuroblastoma varies greatly, ranging from slow-growing tumors that may disappear on their own to highly aggressive forms that spread rapidly to other parts of the body^1,2^.

Bromodomain-containing protein 4 (BRD4) modifies chromatin structure to regulate gene expression. BRD4 belongs to the BET family. One of the most significant BET proteins is the epigenetic reader BRD4, which is implicated in angiogenesis and the emergence of cardiovascular, inflammatory, and central nervous system disorders as well as cancer, notably neuroblastoma^3–7^.

Like all BRD4 members, BD1 and BD2, two highly conserved N-terminal bromodomains (BDs), are present in BRD4. These BDs have the ability to detect acetylated lysine residues and non-histone proteins, which control transcriptionally gene expression, cell division, apoptosis, and cell cycle progression^8,9^. BD1 and BD2 sequences are extremely homologous and conserved. On the other hand, every BRD4 BD helps with protein-specific binding. The two BRD4 BDs may interact with distinct proteins, suggesting that they play distinct biological roles. Compared to BD2, BRD4 BD1 exhibits a greater affinity for the tetra-acetylated histone H4 peptide. Diacetylated histone H3 is recognized by BRD4 BD2, which then binds to it and recruits non-histone proteins.

BRD4 is regarded as a potentially effective therapeutic target for several human diseases^10–16^. According to research, BRD4 is frequently dysregulated in cancers such as neuroblastoma. Dysregulation of BRD4 can lead to abnormal cell growth and proliferation, making it an appealing target for cancer treatment. Therefore, BRD4 inhibitors must be found and developed, and this effort is receiving more and more attention^2^.

Clinical trials involving humans are presently investigating a number of BET inhibitors^10^. An important class of these tiny molecule inhibitors includes azaepine analogues, such (+)-JQ1. The pan-BET family member inhibitor (+)-JQ1, one of the first to be discovered, possessed IC50 values in the nanomolar range^17^. As a result, the pharmacological tool (+)-JQ1 has been extensively employed to explore the biological roles of the BET family in several disorders, including tumor angiogenesis, osteosarcoma, leukemia, and gallbladder cancer^18,19^. Regretfully, the limited selectivity of BET inhibitors within the BET family has limited their applications. According to research, BET family BDs represent exciting new therapeutic targets for the management of obesity, inflammation, cancer, and neurological disorders^20,21^. Thus, several distinct BET inhibitors, including MS436, RVX-208, Olinone, and MS765, have been created to target the BDs of BET family members, particularly. For example, RVX-208 and MS765 are less selective for BRD4 than other members of the BRD family, but they bind to BD2 of BET proteins more readily than BD1 of BET proteins^22,23^. Thus, it is essential to develop powerful and specific BRD4 BD1- and BRD4 BD2-selective compounds with lower affinity for other members of the BET family in order to get a better understanding of the distinct functions and biological activities of each BRD4 BD in a variety of human illnesses.

The purpose of our study is to predict novel BRD4 inhibitors via high-throughput virtual screening, molecular docking, ADMET analysis, and simulations, followed by the evaluation of their potential as therapeutic candidates based on stability and binding energy analysis. Molecular docking is a well-established computational technique used to predict the preferred orientation of a ligand as it binds to a protein receptor, offering insight into binding affinity and specificity^24^. Moreover, Molecular dynamics (MD) simulations assess the stability of the docked complexes under physiological conditions. When combined with ADMET analysis, which assesses the drug likeness, bioavailability, and toxicity, this approach provides a robust framework for in silico drug discovery^25,26^. Recent studies have successfully applied this combination to identify novel therapeutic candidates for various diseases, including cancer and infectious disorders^27–29^. These methods are particularly valuable in early-phase screening, where experimental validation is limited by time and resources.

The field of drug discovery has undergone a paradigm shift in recent years, with computational methods being crucial in the identification of new therapeutic agents^30–33^. This work proposes an integrative pharmacoinformatic approach to identify novel BRD4 inhibitors with potential relevance to neuroblastoma therapy. By combining pharmacophore-based virtual screening, molecular docking, ADMET prediction, and molecular dynamics (MD) simulations, we aim to prioritize compounds with favorable binding, pharmacokinetic, and stability profiles for further preclinical evaluation^27,34^.

## Methodology

### pharmacophore modelling

A chemical template containing the fundamental structural components of physiologically active molecules is known as a pharmacophore model. A pharmacophore model is generated based on the structural characteristics of an active drug and is subsequently processed to perform chemical database screening^35^. The pharmacophore model was created using the chemical structure of the co-crystal ligand and the interactions between the 73B ligand and the BRD4 protein’s (PDB ID: 4BJX) binding pocket via Pharmit web server^36,37^. The server provides a method for screening the chemical databases based on the derived pharmacophoric qualities.

### Pharmacophore-based virtual screening

The virtual screening model was constructed using four pharmacophoric properties of the co-crystal ligand i.e., acceptor, hydrogen bond donor, hydrophobic, and aromatic. The parameters of the virtual screening were determined by applying Lipinski’s rule^38^: hydrogen bond acceptor (HBA) < 10, molecular weight < 500, hydrogen bond donor (HBD) < 5, and logP < 5. The following databases were examined for virtual screening: CHEMBL, Enamine, MCULE, Chemspace, and ChemDiv, with previously reported anticancer activity compounds and a broad range of scaffolds to increase the chances of novel hits.

### Preparation of hit’s pool for docking purposes

The Schrödinger Maestro LigPrep tool was used to optimize, minimize, and prepare the 1089 hits^39^. Conformers were constructed to optimize the shape of each ligand. By adjusting the ligands’ geometry using the OPLS_2005 forcefield, the conformations that are energetically advantageous were guaranteed. The popular scientific forcefield for determining and visualizing the energy of atom-to-atom interactions in molecules is the OPLS_2005 forcefield^40^. Protonation states were generated at pH 7 using Epik. All unfavorable interactions and stretched geometries were eliminated by reducing the compounds’ energy using the conjugate gradient algorithm. The optimized hits with correct ionization states, proper stereochemistry, and minimized geometries were subjected to docking studies.

### Structure-based molecular docking

Molecular docking was performed on the prepared hits against the BRD4 receptor. The BRD4 protein’s crystal structure (PDB ID: 4BJX) was obtained from the PDB database with 1.59 Å resolution, which is the most suitable experimental model for the target protein. The Protein Preparation Wizard was used to prepare the receptor for docking^41^. There were several stages to the receptor’s preparation process. Disulfide bonds were created, metal bonds with zero order were allocated, and bond orders were set. Polar hydrogens were also introduced into the protein structure. Any extra ligands or water molecules were also removed from the crystal structure, and the PROPKA program was used to calculate ionizable groups and the pKa values. At pH 7.0, the hydrogen bonding in the protein was optimized. Finally, the protein structure’s energy was reduced by applying the OPLS_2005 force field^42^. Following protein preparation, a 3D grid was constructed at X, Y, Z coordinates 14.09, 0.72, 9.68 for site-specific docking. The ligands were docked in SP (Standard Precision) mode using the Glide docking module, and 09 poses were generated for each compound^43^. The compounds were examined and selected based on Glide scores.

### Biological activity and drug-likeness analysis

The biological activity, bioavailability scores, and druglike properties of selected hits were predicted via Molinspiration web server (https://www.molinspiration.com/cgi/properties). SwissAdme^44^ and DataWarrior software^45^ respectively. These parameters help to choose which compound can be used for further analysis to get potential leads.

### ADME and toxicity profile analysis

We looked deeper into the ADMET (absorption, distribution, metabolism, excretion, and toxicity) characteristics of the selected docked ligands. These characteristics were forecasted with the QikProp tool from Maestro^46^. Several attributes are estimated by the QikProp tool based on the ligands’ molecular structures. Hydrogen bond acceptors, molecular weight, hydrogen bond donors, QPlogPo/w, QPlogHERG, QPPCaco, QPlogBB, and QPlogKhsa are typical expected features. The logarithm of the octanol-water partition coefficient, which gauges the hydrophobicity and membrane permeability of a compound, is anticipated by QPlogPo/w. QPlogHERG evaluates the likelihood that a ligand will block the hERG potassium channel, which has been associated with potential cardiac toxicity. Using the monolayer of Caco-2 cells as a model for intestinal absorption, QPPCaco calculates a compound’s permeability through the cells. QPlogBB can be used to predict the partition coefficient of the blood-brain barrier, which measures a substance’s ability to penetrate the blood-brain barrier and reach the central nervous system. A vital protein that affects drug distribution and binding in the bloodstream is human serum albumin. The logarithm of this protein’s binding affinity is computed by QPlogKhsa.

### Binding free energy profile of complexes

A computational approach, MM-GBSA, was carried out to investigate binding free energy (BFE) of docked ligands in contact with the targeted receptor^47^. Their efficient binding helps to determine the potential of compounds, i.e., do they function as druglike molecules? For this, MMGBSA module of Prime was employed to estimate the BFE of all protein-ligand complexes. The BFE profile of complexes is a combination of specific components i.e., Coulombic, H-bond, covalent, van der Waals, lipophilic, and solvation interactions. MMGBSA values of complexes were calculated via the Prime tool with the following equations^48^.

1 $$\:\varDelta\:G\text{bind}\:=G\text{complex}-(G\text{protein}+G\text{ligand})$$

2 $$\:\varDelta\:G\text{bind}\:=\varDelta\:E\text{gas}\:+\:\varDelta\:G\text{s}\text{ol}\:-\:T\varDelta\:S$$

3 $$\:\varDelta\:E\text{gas}\:=\varDelta\:E\text{int}\:+\varDelta\:E\text{ELE}\:+\varDelta\:E\text{VDW}$$

4 $$\:\varDelta\:G\text{s}\text{ol}\:=\:\varDelta\:G\text{polar}\:+\:\varDelta\:G\text{nonpolar}\:=\:\varDelta\:G\text{GB}\:+\:\varDelta\:G\text{S}$$

5 $$\:\:T\varDelta\:S\:=\:T(\varDelta\:S\text{trans}\:+\:\varDelta\:S\text{rot}\:+\:\varDelta\:S\text{vib}$$

Where ΔG_bind_ refers to the total binding free energy of complexes in kcal/mol, which is a combination of molecular mechanics gas phase energy (ΔEgas), and solvation free energy (ΔGsol) as displayed in Eq. (2).

### MD simulation study

For the two best compounds, 200 ns Molecular Dynamics simulations were performed using Desmond^49^. Molecular Dynamics simulations were performed to evaluate the ligand and protein complexes’ stability. The stability of complexes was assessed through MD simulations, following a series of steps, including optimization, preprocessing, and minimization. The minimizing procedure was carried out using the OPLS_2005 force field^40^. The compounds underwent solubility in a 10 Å periodic box that contained water molecules of TIP3P^50^. 0.15 M NaCl salt and counter ions were added as needed to neutralize the systems and replicate physiological conditions. The temperature and pressure values for the NPT ensemble were 300 K and 1 atm, respectively. Prior to the simulation initiation, the systems underwent a relaxation phase. Trajectories were recorded and saved at 40 ps intervals during the simulation, enabling subsequent analysis of the results.

### Principal component analysis of potential hits

The principal component analysis (PCA) is a statistical technique utilized to analyze collective and correlated molecular movements of studied complexes. The motions of biological systems were computed and represented in the form of eigenvalues, eigenvectors, and covariance matrices, utilizing proteins’ backbone atoms. These compact motions allow us to investigate conformational changes of the protein in contact with ligand. For this, covariance matrices were formed based on atomic coordinate data of carbon alpha atoms and utilized to obtain corresponding eigenvectors and eigenvalues^51^.

## Results

### Pharmacophore modelling and virtual screening

**Fig. 1:**
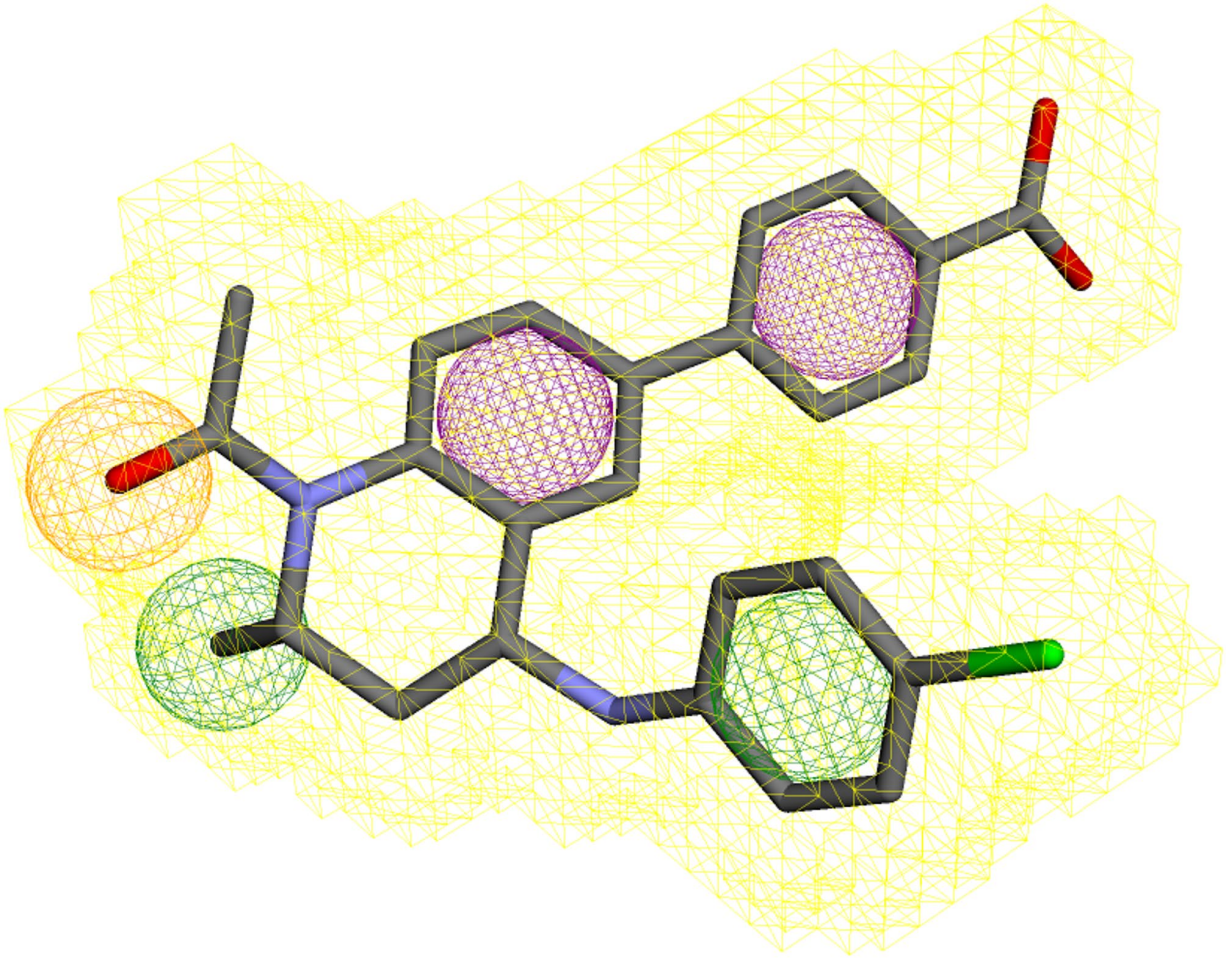
The pharmacophore query model generated by the Pharmit server. Green spheres show the hydrophobic group, purple shows the aromatic ring, while the orange sphere shows the hydrogen bond acceptor.

The pharmacophore query model was created using the pharmacophoric characteristics of the 73B ligand involved in the molecular interactions with the BRD4 protein. Five features (02 hydrophobic, 02 aromatic, and 01 hydrogen bond acceptor) were used to generate the query model (Fig. 1). The X, Y, and Z coordinates of the features are shown in Table 1.

**Table 1:** The pharmacophoric features and their coordinates were generated by the pharmit server.

Pharmacophoric Features	X	Y	Z	Radius
Aromatic	13.99	1.86	10.84	1
Aromatic	15.41	-2.02	11.9	1
Hydrogen Acceptor	11.75	6.16	10.33	1
Hydrophobic	13.17	0.01	5.85	1
Hydrophobic	15.03	6.02	10.0	1

Based on these features, pharmacophore-based virtual screening of five databases was conducted, and the hits meeting the screening criteria were selected (Table 2). A total of 1089 hits were collectively obtained from all screened databases. Among these hits, the MCULE database produced the highest number of hits.

**Table 2:** The generated hits from each database are based on pharmacophore-based virtual screening.

Sr.	Databases	Hits
1	CHEMBL	35
2	ChemDiv	22
3	Chemspace	52
4	Enamine	12
5	MCULE	968
	Total	1089

### Structure-based molecular docking

The 1089 hit compounds were selected after virtual screening and docked to the BRD4 receptor to predict the binding affinities by using the standard precision mode of the Glide tool. Based on the binding affinities, the top ten compounds were selected for further analysis. The chosen compounds’ binding affinities fell within − 9.623 to -8.894 kcal/mol range. The binding affinities for the selected compounds indicated that these have the probability of inhibiting the function of BRD4. The BIOVIA Discovery Studio tool was utilized to analyze the molecular interactions between the chosen hits and the binding pocket of the BRD4 receptor. The observed interactions mainly involved: hydrogen bonds, van der Waal interactions, Pi-Pi Stacked, Pi-Sulfur, Pi-Sigma, and Alkyl interactions. The binding affinities and docking scores of each of the best candidate compounds are largely determined by these interactions. Notably, the overall strength of the resulting complex is significantly influenced by the creation of hydrogen bonds between the ligand and the amino acid inside the active regions of molecules. Consequently, these interactions consistently enhance the docking results^52^.

#### Conformational exploration of binding poses

**Fig. 2:**
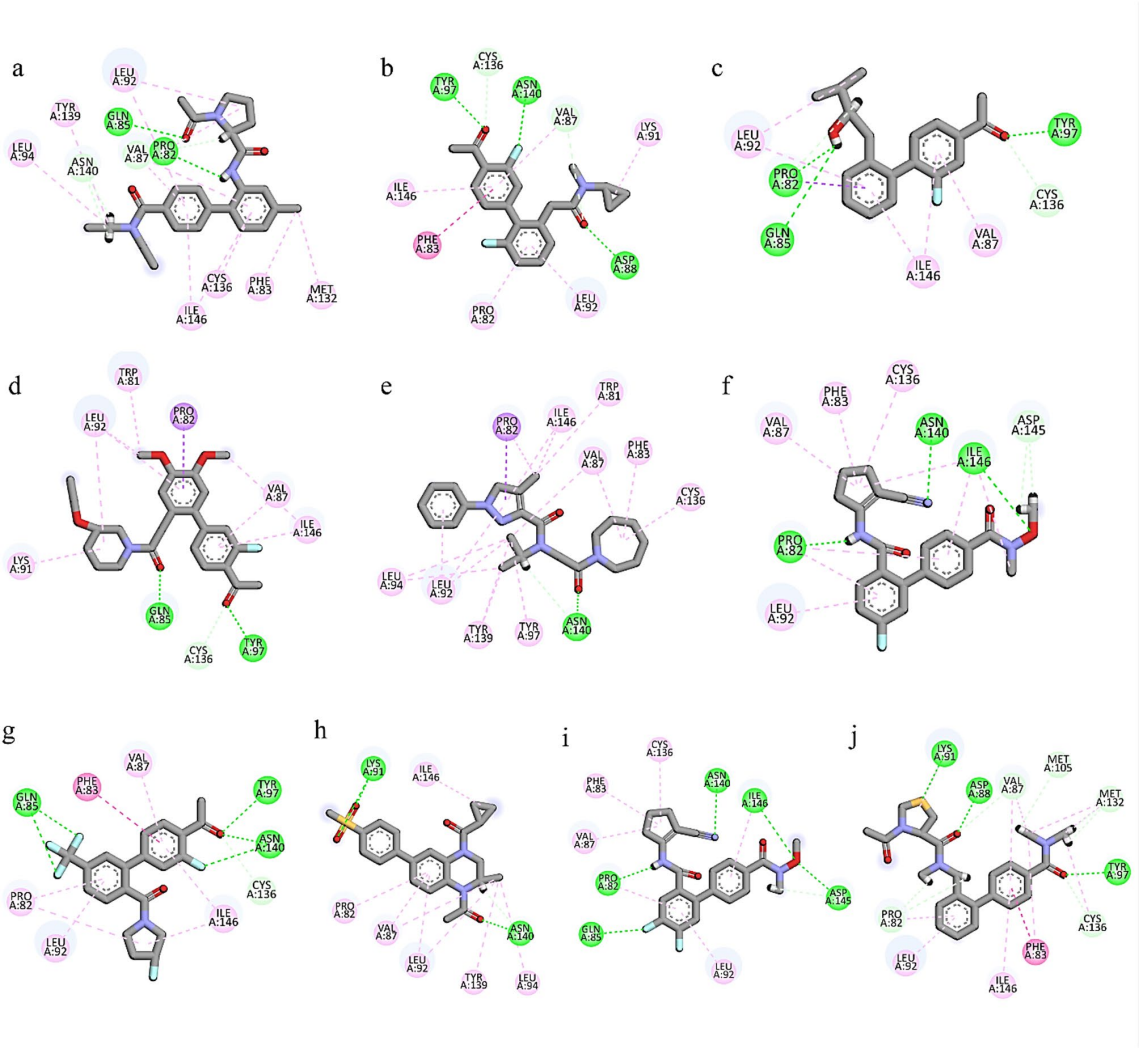
The molecular interactions of the compounds. (**a**) MCULE-3,895,595,807, (**b**) MCULE-8,812,795,806, (**c**) CSC101378549, (**d**) MCULE-6,216,741,487, (**e**) CSC101661920, (**f**) MCULE-3,872,683,406, (**g**) MCULE-2,532,030,345, (**h**) CHEMBL4216188, (**i**) MCULE-6,796,614,739, (**j**) MCULE-1,485,100,159. Green spheres show hydrogen bonds, purple shows Pi-Sigma interaction, and magenta shows alkyl interactions.

**Fig. 3:**
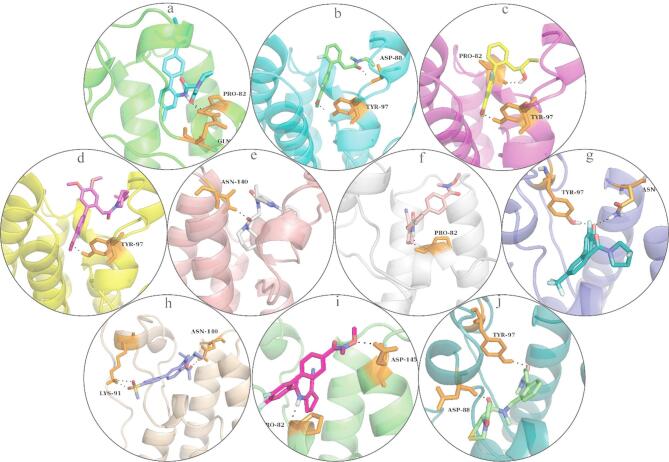
The Binding modes of the docked compounds. (**a**) MCULE-3,895,595,807, (**b**) MCULE-8,812,795,806, (**c**) CSC101378549, (**d**) MCULE-6,216,741,487, (**e**) CSC101661920, (**f**) MCULE-3,872,683,406, (**g**) MCULE-2,532,030,345, (**h**) CHEMBL4216188, (**i**) MCULE-6,796,614,739, (**j**) MCULE-1,485,100,159.

**MCULE-3,895,595,807** formed four hydrogen bonds with Gln85, Pro82, Val87, Asn140, and seven alkyl interactions with Leu94, Tyr139, Leu92, Met132, Phe83, Cys136, Ile146 residues (Fig. 2a). **MCULE-8,812,795,806** formed five hydrogen bonds with Tyr97, Asn140, Asp88, Val87, Cys136, and five alkyl interactions with Ile146, Phe83, Pro82, Leu92, Lys91 (Fig. 2b). **CSC101378549** formed four hydrogen bonds with Pro82, Gln85, Tyr97, Cys136 and the three alkyl interactions with the Leu92, Ile146, Val87 (Fig. 2c). **MCULE-6,216,741,487** formed three hydrogen bonds with Gln85, Tyr97, Cys136, one Pi-Sigma interaction with Pro82, and five alkyl interactions with Lys91, Leu92, Trp81, Pro82, Val87, Ile146 (Fig. 2d). **CSC101661920** formed one hydrogen bond with Asn140, one Pi-Sigma interaction with Pro82, and nine alkyl interactions with Ile146, Trp81, Val87, Phe83, Cys136, Tyr97, Tyr139, Leu92, Leu94 (Fig. 2e). **MCULE-3,872,683,406** formed four hydrogen bonds with Pro82, Asn140, Ile146, Asp145, and four alkyl interactions with Leu92, Val87, Phe83, Cys136 (Fig. 2f). **MCULE-2,532,030,345** formed four hydrogen bonds with Gln85, Tyr97, Asn140, Cys136, and five alkyl interactions with Phe83, Val87, Ile146, Leu92, Pro82 (Fig. 2g). **CHEMBL4216188** formed two hydrogen bonds with Lys91, Asn149, and six alkyl interactions with Ile46, Pro82, Val87, Leu92, Tyr139, Leu194 (Fig. 2h). **MCULE-6,796,614,739** formed five hydrogen bonds with Gln85, Pro82, Asn140, Ile146, Asp145, and four alkyl interactions with Val87, Phe83, Cys136, Leu92 (Fig. 2i). **MCULE-1,485,100,159** formed seven hydrogen bonds with Lys91, Asp88, Val87, Met105, Met132, Cys136, Pro82, and three alkyl interactions with Leu92, Ile146, Phe83 (Fig. 2j). The docked compounds’ binding modes are shown in Fig. 3. The docking scores, the hydrophobic interaction, and hydrogen bond lengths are shown in Table 3. Upon comparison with the known pharmaceutical agents, such as RVX-208 and MS436, which depict similar H-bond interactions with Asn140, Asn135, and Pro82 residues. Similar interacting residues in the active site exhibit analogous binding strengths. Besides this, the selected compounds show lower binding energy than known pharmaceutical agents, which indicates stronger protein-ligand interactions.

**Table 3:** The selected compounds’ binding affinity as well as their molecular interactions.

Sr.	Compound code	Glide score (kcal/mol)	Hydrogen Bonding	Hydrophobic Interactions
1	MCULE-3,895,595,807	− 9.623	Gln85(2.14), Pro82(1.98), Val87(2.52), Asn140(2.25)	Leu94(5.17), Tyr139(5.13), Leu92(4.56), Met132(4.50), Phe83(4.61), Cys136(4.67), Ile146(4.47)
2	MCULE-8,812,795,806	− 9.49	Tyr97(2.05), Asn140(2.23), Asp88(1.99), Val87(2.80), Cys136(2.46)	Ile146(4.72), Phe83(5.73), Pro82(5.07), Leu92(4.60), Lys91(5.15)
3	CSC101378549	− 9.405	Pro82(2.24), Gln85(2.97), Tyr97(1.88), Cys136(2.50)	Leu92(4.29), Ile146(5.14), Val87(4.09)
4	MCULE-6,216,741,487	− 9.325	Gln85(1.66), Tyr97(1.92), Cys136(2.34)	Lys91(4.66), Leu92(5.18), Trp81(4.97), Pro82(2.92), Val87(3.87), Ile146(4.83)
5	CSC101661920	− 9.114	Asn140(2.03)	Pro82(2.79), Ile146(4.25), Trp81(4.98), Val87(4.79), Phe83(4.25), Cys136(4.65), Tyr97(4.45), Tyr139(5.32), Leu92(4.88), Leu94(4.54)
6	MCULE-3,872,683,406	− 9.064	Pro82(2.05), Asn140(2.20), Ile146(2.75), Asp145(2.66)	Leu92(4.66), Val87(4.33), Phe83(4.49), Cys136(4.97)
7	MCULE-2,532,030,345	− 9.036	Gln85(2.35), Tyr97(2.32), Asn140(2.47), Cys136(2.51)	Phe83(5.86), Val87(4.02), Ile146(4.09), Leu92(4.58), Pro82(4.99)
8	CHEMBL4216188	− 8.933	Lys91(2.08), Asn149(1.76)	Ile46(4.94), Pro82(5.08), Val87(4.50), Leu92(4.98), Tyr139(5.34), Leu194(4.99)
9	MCULE-6,796,614,739	− 8.913	Gln85(2.57), Pro82(2.11), Asn140(2.10), Ile146(2.81), Asp145(2.68)	Val87(4.29), Phe83(4.50), Cys136(5.03), Leu92(4.80)
10	MCULE-1,485,100,159	− 8.894	Lys91(2.57), Asp88(1.83), Val87(2.83), Met105(2.83), Met132(2.45), Cys136(2.40), Pro82(2.31)	Leu92(4.60), Ile146(4.68), Phe83(5.68)

### Prediction of biological activity, availability, and drug-likeness of top ten hits

Biological activity of top hits was determined against human receptors that can act as drug targets. In Table 4, bioactivity scores of chemicals were calculated for certain parameters i.e., GPCR ligand, enzyme inhibition, kinase, and protease inhibition. The biological activity scores considered significant above 0.0, moderate ranged between − 5 and 0.0, while below − 5 were considered inactive^53^. The top 10 chemicals’ bioactivity scores, ranging from 0.68 to −  0.48, clearly reveal that they have medicinal potential. MCULE-2,532,030,345 and CSC101378549 hits displayed significant results within the active range, depicting a low risk of undesirable behavior i.e., mutagenicity or poor absorption, and implied potential drug-like behavior^54^.

**Table 4:** Biological activity scores of the top 10 hits with certain human receptors.

Parameters of Bioactivity Score						
Compounds	GPCR ligand	Ion channel Modulator	Kinase Inhibitor	Nuclear Receptor ligand	Protease Inhibitor	Enzyme Inhibitor
MCULE-3,895,595,807	0.13	− 0.13	− 0.01	− 0.24	0.20	− 0.08
MCULE-8,812,795,806	0.13	0.00	− 0.08	− 0.28	0.07	0.07
**CSC101378549**	**0.18**	**0.15**	**0.13**	**0.34**	**0.12**	**0.16**
MCULE-6,216,741,487	0.07	− 0.08	− 0.13	− 0.12	− 0.01	− 0.03
CSC101661920	0.12	− 0.20	− 0.17	− 0.48	− 0.04	− 0.12
MCULE-3,872,683,406	− 0.02	− 0.39	− 0.21	− 0.33	− 0.07	− 0.02
**MCULE-2,532,030,345**	**0.54**	**0.16**	**0.11**	**0.40**	**0.68**	**0.31**
MCULE-6,216,741,487	0.07	− 0.08	− 0.13	− 0.12	− 0.01	− 0.03
CHEMBL4216188	0.10	− 0.16	− 0.07	− 0.02	0.21	0.13
MCULE-6,796,614,739	− 0.01	− 0.36	− 0.21	− 0.30	− 0.01	− 0.00

Significant values are in bold.

All hits lie within the range of drug-likeness (−  10 to + 10) criteria, while MCULE-3,895,595,807, MCULE-8,812,795,806, CSC101661920, MCULE-2,532,030,345, and CHEMBL4216188 hits are considered as orally active drugs compared to others. They have lower chances of rejection in the drug development process and possess better pharmacokinetic properties. All compounds possess 0.55 bioavailability scores, have significant absorption, and are preferred for oral delivery^55^. Some of the compounds depict mutagenicity, tumorigenicity, and irritant effects, while none show reproductive effects, as shown in Table 5.

**Table 5:** Drug-likeness, bioavailability, and other properties of top hits.

Compounds	Drug-likeness	Mutagenic	Tumorigenic	Reproductive Effective	Irritant	Bioavailability
MCULE-3,895,595,807	8.3741	none	none	none	high	0.55
MCULE-8,812,795,806	1.4053	none	none	none	none	0.55
CSC101378549	− 2.4578	none	none	none	none	0.55
MCULE-6,216,741,487	− 2.9803	none	none	none	none	0.55
CSC101661920	1.3073	none	none	none	low	0.55
MCULE-3,872,683,406	− 4.6495	high	none	none	none	0.55
MCULE-2,532,030,345	3.2117	none	none	none	none	0.55
MCULE-6,216,741,487	− 2.9803	none	none	none	none	0.55
CHEMBL4216188	6.8838	high	high	none	none	0.55
MCULE-6,796,614,739	− 4.6495	high	none	none	none	0.55

### ADME and toxicological properties of top hits

The preferred compounds’ ADMET (absorption, distribution, metabolism, excretion, and toxicity) characteristics were examined. The compounds’ ADMET characteristics range within permissible bounds. “QPlogPo/w” (−  2.0 to 6.5), “QPlogHERG” (<−  5), “QPlogBB” (−  3.0 to 1.2), “QPPCaco” (< 25 poor, > 500 great), and “QPlogKhsa” (−  1.5 to 1.5) were the cutoff values for the ADMET parameters. The Compounds’ ADMET and physicochemical properties are shown in Table 6. Upon analysis of the ADMET properties, it was observed that only **CSC101378549** and **MCULE-2,532,030,345** compounds met the selection criteria, so these two compounds were subjected to binding pose analysis and MD simulations for stability analysis.

**Table 6:** The chosen compounds’ physicochemical and ADMET characteristics.

Compounds	MW	HBD	HBA	QPlogPo/w	QPlogHERG	QPPCaco	QPlogBB	QPlogKhsa
MCULE-3,895,595,807	421.538	1	8	3.469	− 4.39	917.017	− 0.707	0.126
MCULE-8,812,795,806	343.372	0	5	3.297	− 3.944	871.76	− 0.42	− 0.058
**CSC101378549**	**300.372**	**1**	**3**	**4.136**	**− 5.17**	**1486.451**	**− 0.54**	**0.452**
MCULE-6,216,741,487	443.514	0	8	3.512	− 4.309	791.788	− 0.836	− 0.14
CSC101661920	382.505	0	7	3.58	− 4.13	3200.299	− 0.079	− 0.03
MCULE-3,872,683,406	393.417	1	8	3.306	− 6.217	582.041	− 1.107	0.145
**MCULE-2,532,030,345**	**397.344**	**0**	**5**	**4.632**	**− 5.239**	**1991.902**	**0.108**	**0.439**
MCULE-6,216,741,487	443.514	0	8	3.798	− 4.67	801.161	− 0.824	− 0.05
CHEMBL4216188	412.503	0	10	2.063	− 5.644	429.515	− 1.127	− 0.433
MCULE-6,796,614,739	412.503	0	10	2.063	− 5.644	429.515	− 1.127	− 0.433

Significant values are in bold.

“QPlogHERG” (<−  5), “QPlogPo/w” (−  2.0 to 6.5), “QPlogBB” (−  3.0 to 1.2), “QPPCaco” (< 25 poor, > 500 great), and “QPlogKhsa” (−  1.5 to 1.5).

### Binding free energy calculations of docked complexes

The binding energy of ligands to protein molecules is commonly measured using the MM/GBSA technique^56^. The BRD4 complex’s binding free energy and the impact of extra non-bonded interaction energies were assessed (Table 7). The binding free energy of MCULE-2,532,030,345 was − 86.300 kcal/mol, whereas that of CSC101378549 to BRD4 was − 82.851 kcal/mol, indicating stronger binders than others. $\:\varDelta\:$G_bind_ is governed by non-bonded interactions such as $\:\varDelta\:$G_bind_ Coulomb, $\:\varDelta\:$G_bind_ Packing, $\:\varDelta\:$G_bind_ Hbond, $\:\varDelta\:$G_bind_Lipo, and $\:\varDelta\:$G_bind_vdW. The average binding energy was most affected by the $\:\varDelta\:$G_bind_Lipo, $\:\varDelta\:$G_bind_vdW, and $\:\varDelta\:$G_bind_Coulomb energies among all interaction types, representing polar interactions and a better hydrophobic fit. The hydrophobicity of compounds is due to the nonpolar solvation interactions, which depict good binding of chemicals with the targeted receptor^32,33^. Conversely, the final average binding energies were least affected by the $\:\varDelta\:$G_bind_Solv_GB, $\:\varDelta\:$G_bind_Covalent, and strain energies, demonstrating less conformational distortion. Furthermore, BRD4 complexes showed stable hydrogen bonds with amino acid residues based on their G_bind_Hbond interaction values. Thus, the binding energy obtained from the docking data was well-supported by the MM-GBSA estimations^57^.

**Table 7:** Calculations of the binding free energy of 10 potential compounds in contact with targeted receptor BRD4.

Compounds	Bind	Coulomb	Covalent	Hbond	Lipo	vdW	S_Energy	Solv_GB
MCULE-3,895,595,807	− 68.971	− 4.4183	2.9296	− 0.5201	− 46.968	− 38.247	11.175	19.339
MCULE-8,812,795,806	− 78.077	− 12.666	3.4081	− 0.4714	− 36.888	− 38.382	6.1409	7.541
**CSC101378549**	**− 82.851**	**− 13.054**	**3.8177**	**− 0.7423**	**− 39.129**	**− 34.081**	**10.5781**	**8.456**
MCULE-6,216,741,487	− 66.769	− 9.3676	15.131	− 0.5098	− 42.670	− 41.714	22.6154	12.877
CSC101661920	− 70.669	− 2.6130	20.029	− 0.2475	− 57.525	− 37.162	36.7824	7.163
MCULE-3,872,683,406	− 79.671	− 12.002	4.1067	− 0.7324	− 44.647	− 42.788	7.04354	9.451
**MCULE-2,532,030,345**	**− 86.300**	**− 12.208**	**5.0302**	**− 0.2461**	**− 42.066**	**− 41.943**	**11.6989**	**7.399**
MCULE-6,216,741,487	− 66.769	− 9.3676	15.131	− 0.5098	− 42.670	− 41.714	22.6154	12.877
CHEMBL4216188	− 79.168	− 13.451	3.4976	− 1.2082	− 42.326	− 42.719	7.72876	17.257
MCULE-6,796,614,739	− 79.939	− 7.2884	1.5828	− 0.7358	− 41.073	− 40.225	11.0206	6.875

Significant values are in bold.

### Binding pose analysis of potent complexes

**Fig. 4:**
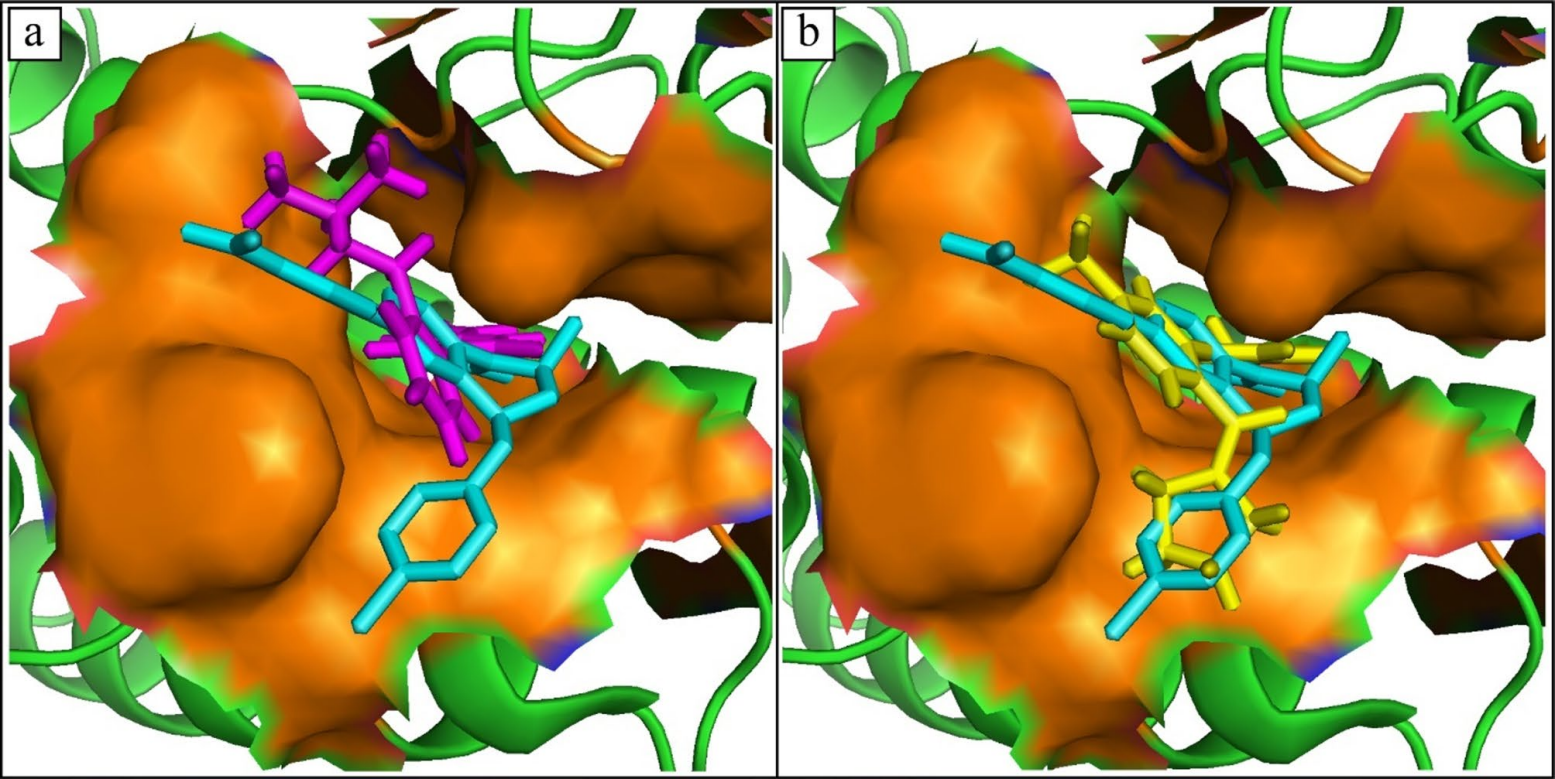
The plausible binding modes of the best two compounds aligned on the co-crystal ligand (cyan sticks). (**A**) CSC101378549 (magenta sticks), (**B**) MCULE-2,532,030,345 (yellow sticks).

The two chemicals chosen for further examination were positioned onto the co-crystal ligand to explore their potential binding mechanisms after examining their molecular interactions. The analysis revealed that the docked compounds were positioned onto the co-crystal ligand in the same active site and showed a similar binding mode (Fig. 4). Thus, the possible binding mechanisms of the selected compounds were subjected to stability analysis by employing the MD Simulation study.

### Structure stability analysis of potential complexes

#### RMSD

**Fig. 5:**
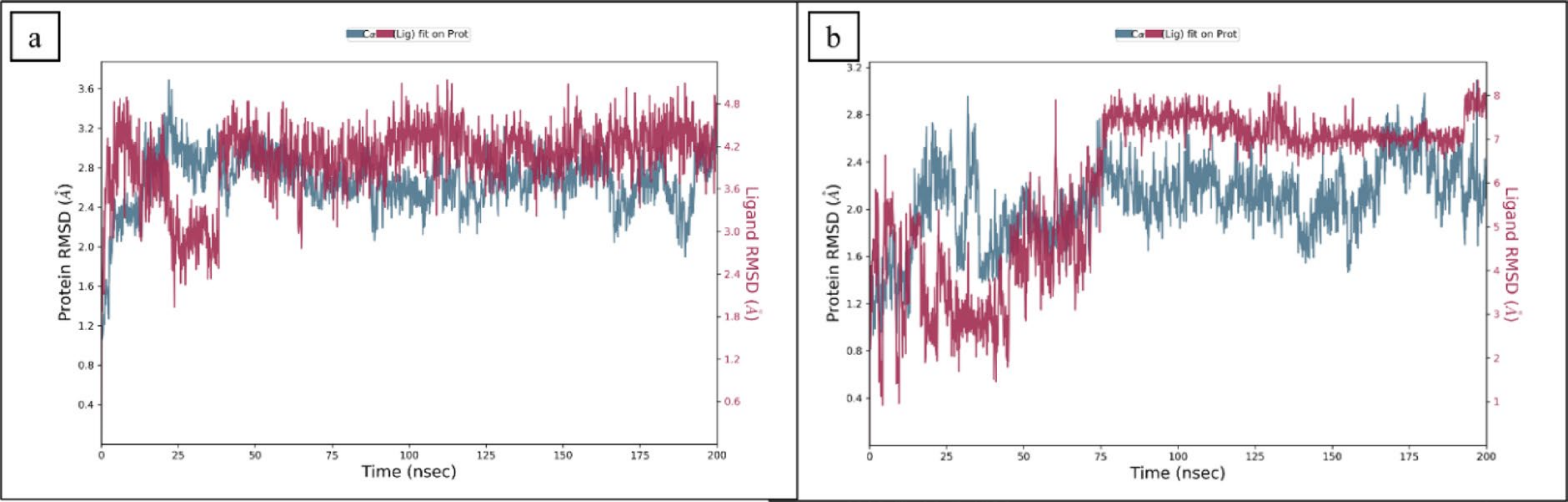
The RMSD of BRD4 complexes calculated during the 200 ns simulation. (**A**) CSC101378549, (**B**) MCULE-2,532,030,345.

The stability of the chosen compounds against the BRD4 receptor was verified using 200 ns molecular dynamics (MD) simulations. The root mean square deviation (RMSD) of the carbon alpha atoms was calculated to investigate the complexes’ overall structural variations and alterations over the simulation^58^. The RMSD values for the **CSC101378549** progressively elevated to ~ 3.2 Å till 25 ns and then showed a decrease in RMSD value of about 2.1 Å up to 40 ns. After 40 ns, it increased again and reached ~ 3.0 Å. Following that, it stabilized in this range until the simulation’s end, while the ligand’s RMSD completely lined up with the protein’s RMSD. (Fig. 5a). However, the RMSD of **MCULE-2,532,030,345** was lower than the RMSD of the protein till half of the simulation time and depicts more fluctuations than CSC101378549 within the acceptable range. It started at a range of ~ 1.6 Å at 5 ns, and stayed there until 75 ns with minor escalation, which increased to 2.0 Å. However, at 80 ns, it reached stability at 2.8 Å towards the end of the simulation (Fig. 5b). After equilibrium, no clear fluctuations were observed, which indicates that both compounds remained bound in the active site of the targeted receptor.

#### RMSF

**Fig. 6:**
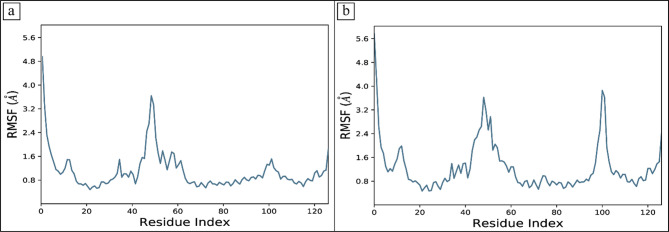
The residual fluctuations of the BRD4 receptor upon binding of the selected compounds. (**a**) CSC101378549, (**b**) MCULE-2,532,030,345.

**Fig. 7:**
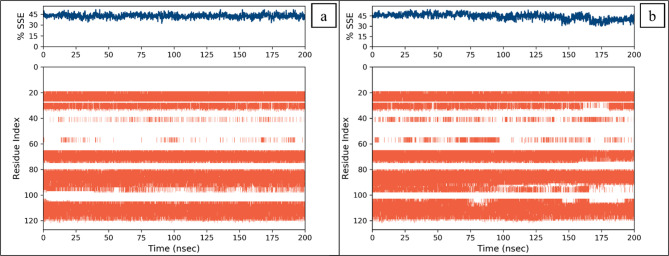
The percentage of secondary structure elements of BRD4 receptor upon binding of the selected compounds. (**a**) CSC101378549, (**b**) MCULE-2,532,030,345.

Root mean square fluctuations (RMSF) values were computed in order to study the dynamic behavior of the proteins while they were bound to the ligands^59^. For each protein residue over the simulation period, RMSF values give detailed information on the residue’s mobility and flexibility. Based on the expected RMSF values, most protein residues changed very slightly during the simulation, which was less than 2Å. This suggests that these residues maintained relative stability and stiffness while the ligands were present. However, the RMSF values were higher in the protein’s loop regions, which were around 4Å^60^. According to the RMSF analysis, most residues in the protein-ligand complexes held onto their rigid forms, causing the complex to stay stable. RMSF values of loop regions were higher, indicating that these areas displayed larger variations than others and could have engaged in dynamic interactions with the ligands (Fig. 6). Most protein residues showed relatively slight changes, but loop parts showed significantly higher levels of flexibility (Fig. 7). The blue regions revealed the presence of alpha helices, while the orange color indicates the beta sheets. The loops were exhibited in white. During the simulation, it was estimated that the secondary structures did not show fluctuations and remained stable upon binding the ligands.

#### Protein-Ligand contacts

**Fig. 8:**
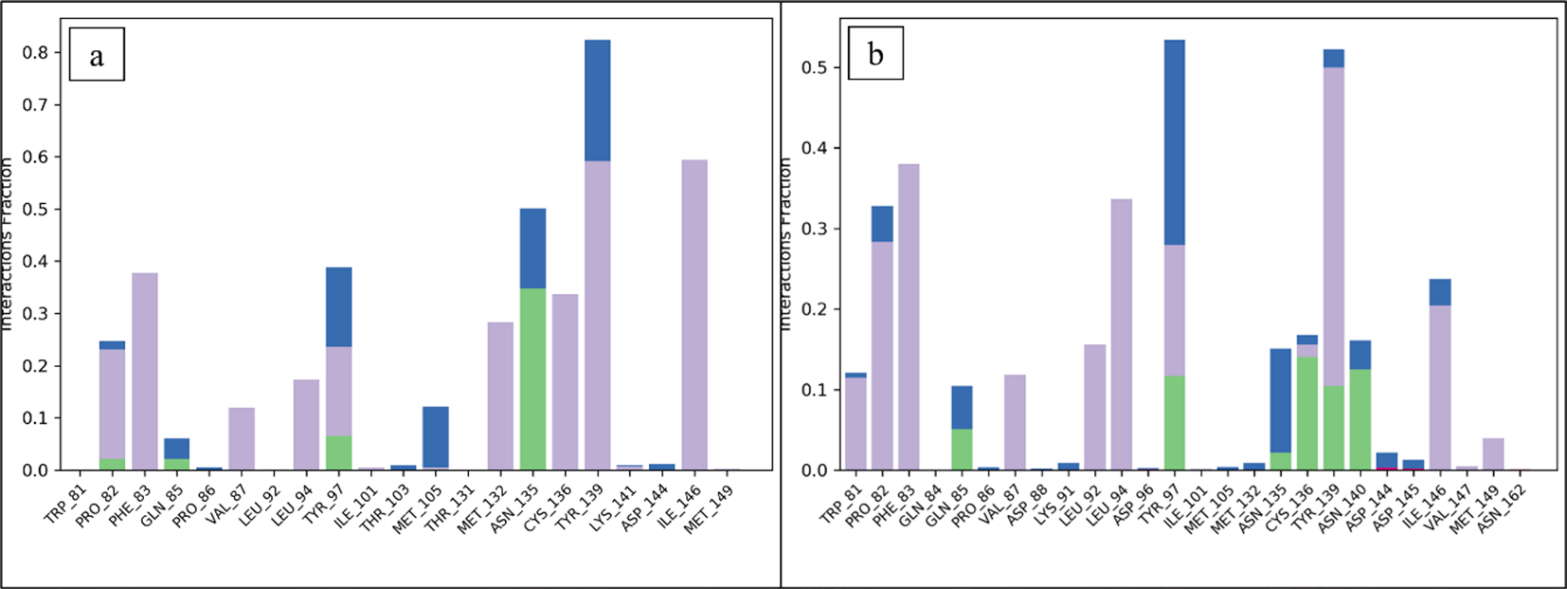
Protein-ligand interaction during MDS. (**a**) CSC101378549 and (**b**) MCULE-2,532,030,345. Large, stacked bars represent the interacting residues. Green represents H-bonds, grey for hydrophobic interactions, blue for water bridges, and pink for ionic interactions.

Based on the MD Simulation study, the most important types of interactions between the ligands and the protein were identified as hydrophobic, hydrogen, and ionic bonds. The functional properties of the protein-ligand complexes are stabilized and regulated by these interactions^34^. **CSC101378549** participated in the hydrogen bonding with the residues Pro82, Gln85, Tyr97, and Asn135. Met105, and Tyr139 residues show water bridges while Phe83, Val87, Leu94, Met134, Cys136, Ile146 depicts hydrophobic interactions (Fig. 8a). In the complex of **MCULE-2,532,030,345**, residues that form bonds with hydrogen were Gln85, Tyr97, Asn135, Cys136, Tyr139, and Asn140 while Phe83, Val87, Leu92, Leu94 and Tyr139 form hydrophobic interactions (Fig. 8b). The interactions between hydrogen bonds that were seen in the MD simulations not only highlighted the specific residues that were crucial for stabilizing the protein-ligand complexes, but they also provided insight into the crucial interactions that maintain the complexes’ general stability and binding affinity^61^.

#### hydrogen bonding analysis

**Fig. 9:**
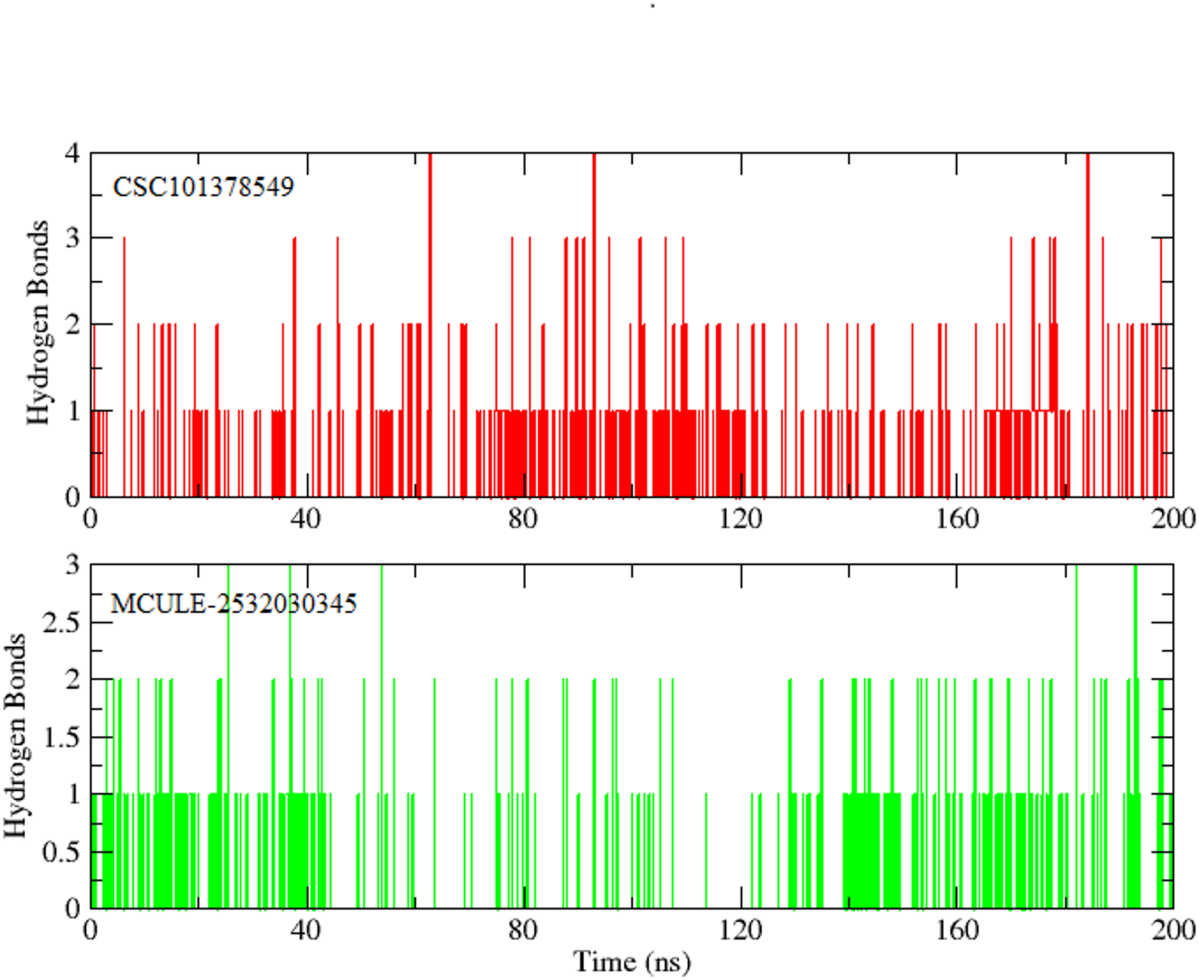
The number of hydrogen bonds between BRD4 and selected ligands (CSC101378549 and MCULE-2532030345) calculated during a 200 ns simulation.

Hydrogen bonding is essential for the protein-ligand combination to remain stable. Consequently, the ligand hydrogen bonds and active site residues were calculated. The hydrogen bonding plots indicate that **CSC101378549** made at least 02 hydrogen bond with the protein. The number of hydrogen bonds exceeded 03 and 04 at some frames (Fig. 9a). On the other hand, **MCULE-2,532,030,345** made up to 03 hydrogen bonds till 50 ns, and then the number of hydrogen bonds reduced to 02 in the later part of the simulation. Once again, 03 H-bonds can be seen at the end of the simulation (Fig. 9b).

#### SASA and radius of gyration

**Fig. 10:**
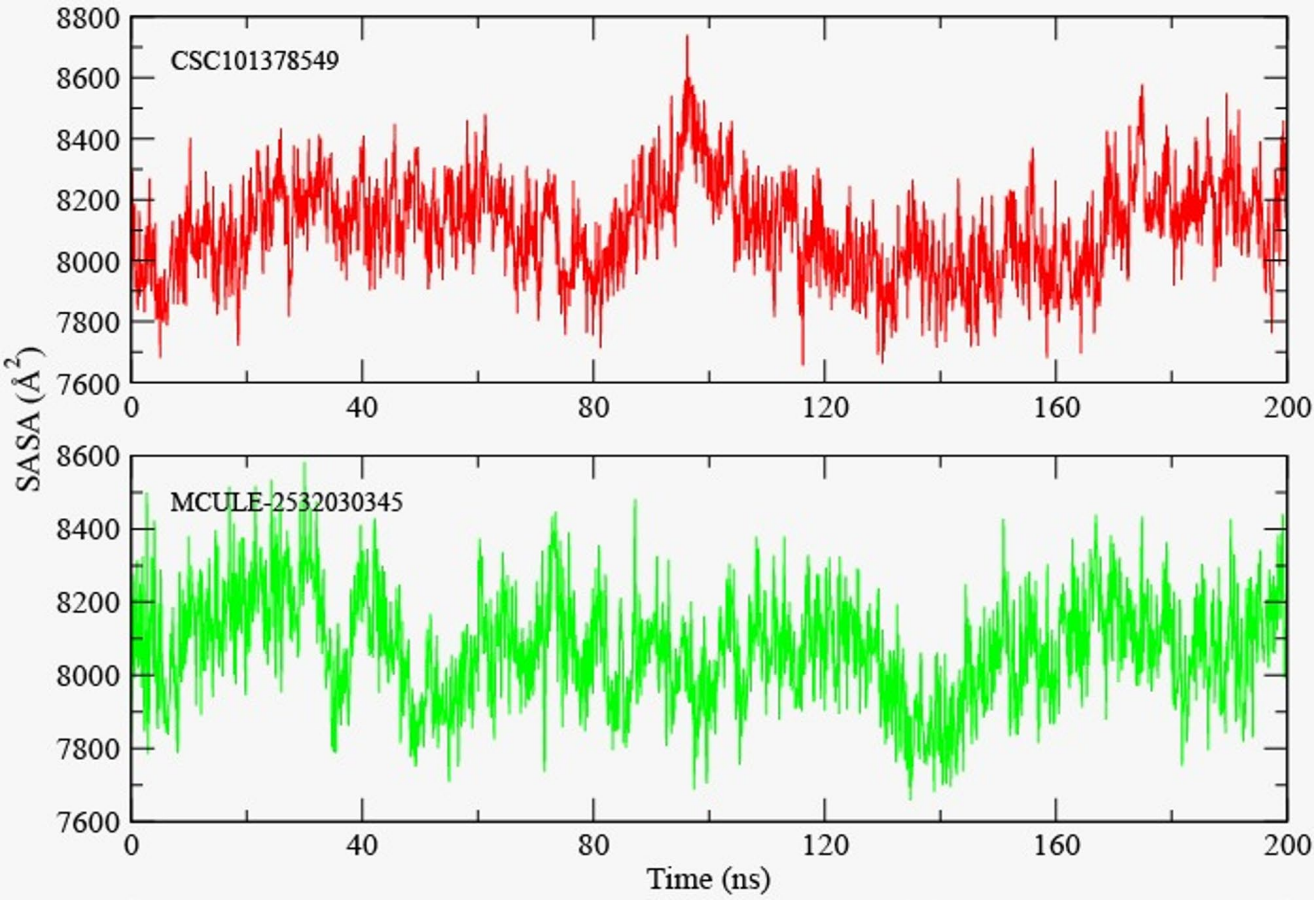
Solvent accessible surface area (SASA) for 200ns molecular dynamic simulations. (**A**) CSC101378549 and (**B**) MCULE-2,532,030,345.

Solvent accessible surface area (SASA) demonstrates protein-ligand complex accessibility to solvent and interactions with solvent. SASA also gives insights regarding protein folding and stability events, along with conformational changes associated with ligand binding^62^. Moreover, SASA impacts drug absorption and metabolism. The average SASA values for CSC101378549-BRD4 complex were 8200 ± 200 Å^2^, and for MCULE-2,532,030,345-BRD4 system lie within the acceptable range of 7800 to 8400 Å^2^, which indicates that the ligand stays stable inside the target protein’s binding pocket and protein remains compact over the simulation time (Fig. 10).

**Fig. 11:**
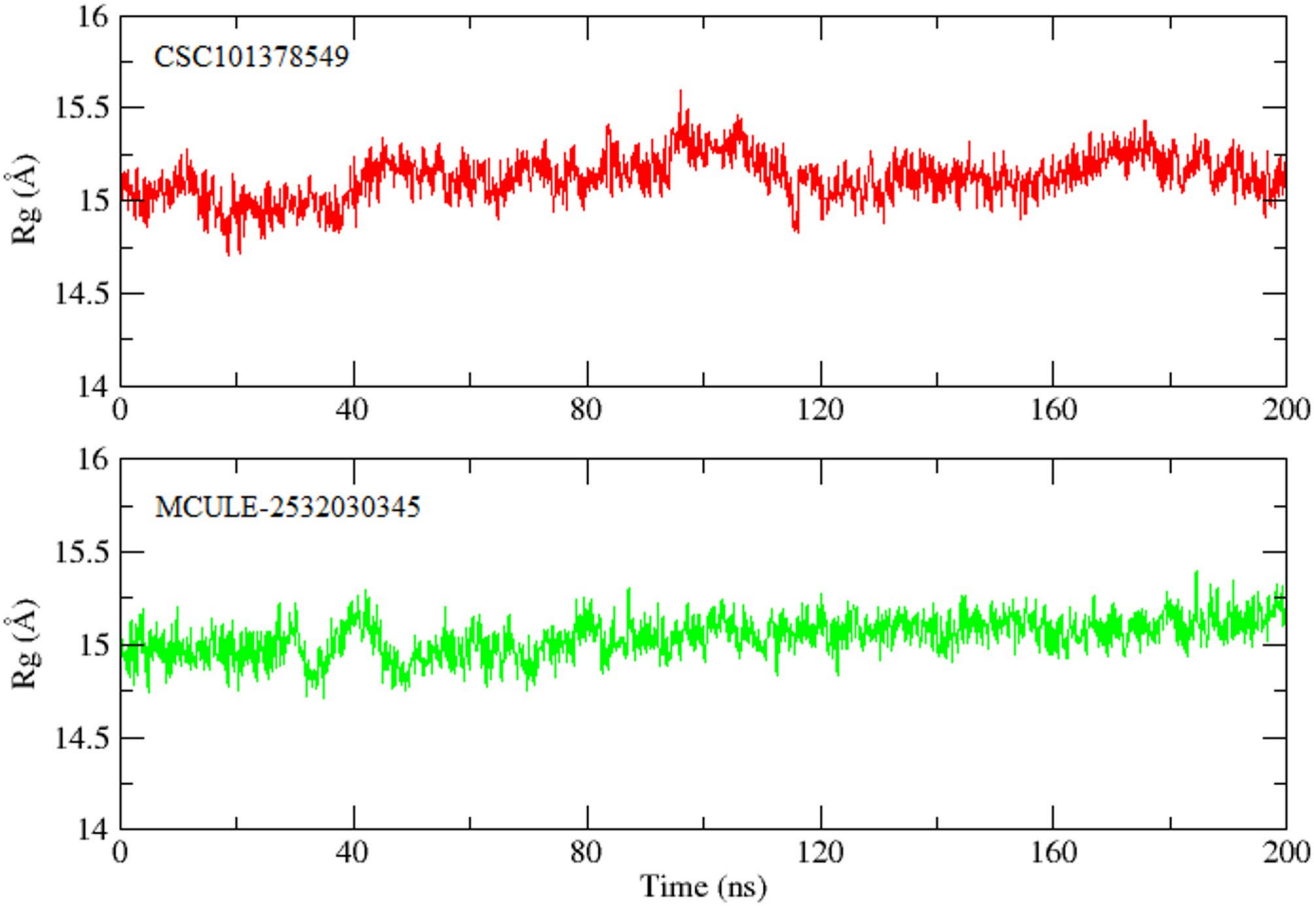
Radius of Gyration (rGyr.) for 200ns MD Simulation. (**A**) CSC101378549 and (**B**) MCULE-2,532,030,345.

The radius of gyration (Rg), used to track compactness and structural arrangement (folding) of protein^63^. Figure 11 demonstrates fluctuations in Rg of CSC101378549-BRD4 and MCULE-2,532,030,345-BRD4 systems over the simulation time. The average Rg values were 15 ± 0.5 Å^2^ and 15 ± 0.3 Å^2^ for CSC101378549-BRD4 and MCULE-2,532,030,345-BRD4 systems, respectively. CSC101378549-BRD4 complex depicts no sharp and sudden peaks, while MCULE-2,532,030,345-BRD4 system demonstrates inevitable fluctuation at 40ns. According to these findings, Rg values of both complexes varied within the permitted range, and the receptor remained compact during the simulation time.

### Principal component analysis

**Fig. 12:**
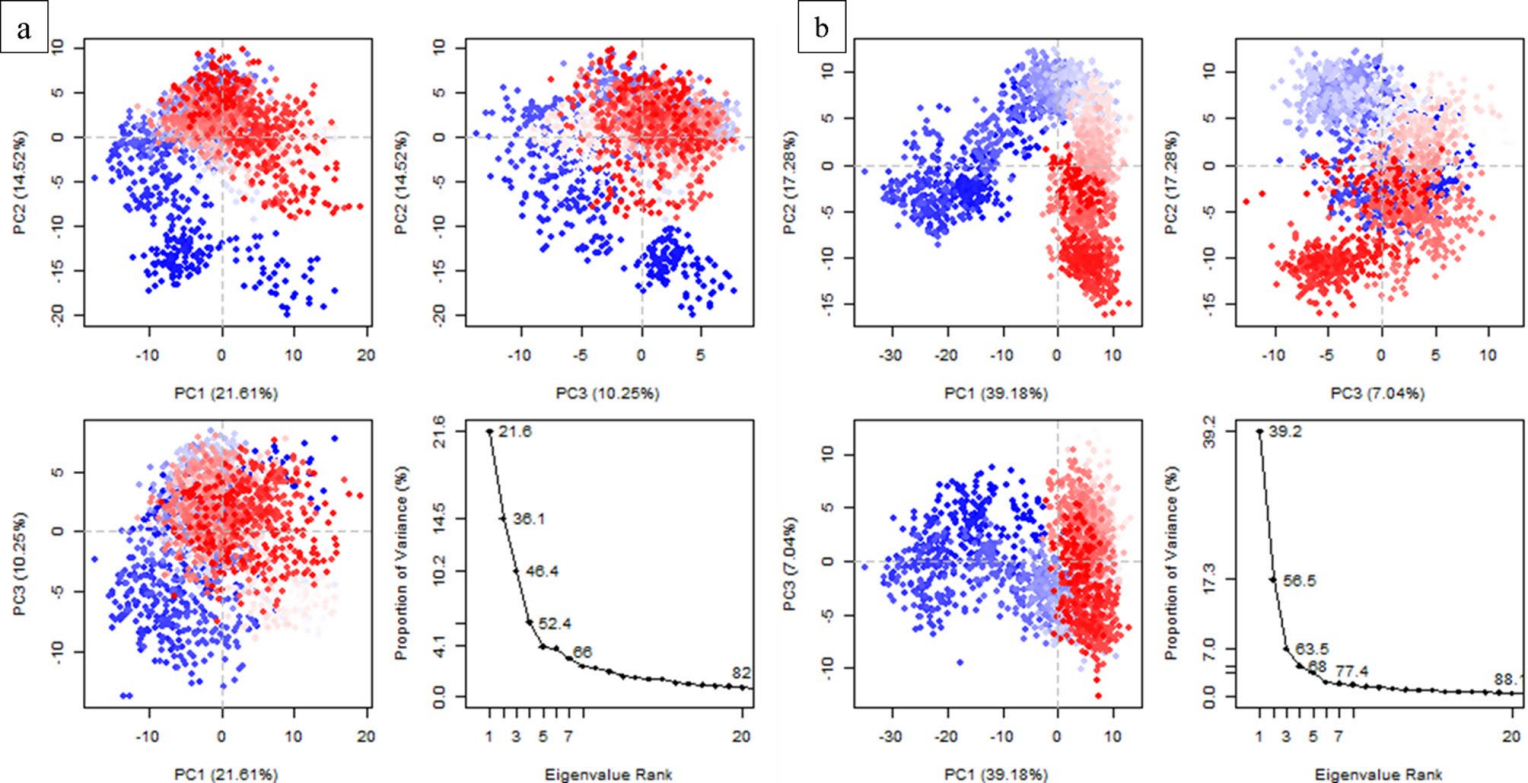
The principal component analysis indicates fluctuations in different hyperspaces. (**a**) CSC101378549 and (**b**) MCULE-2,532,030,345. The Blue regions showed the most significant movement, the white regions showed intermediate movement, while the red regions showed less flexibility.

**Fig. 13:**
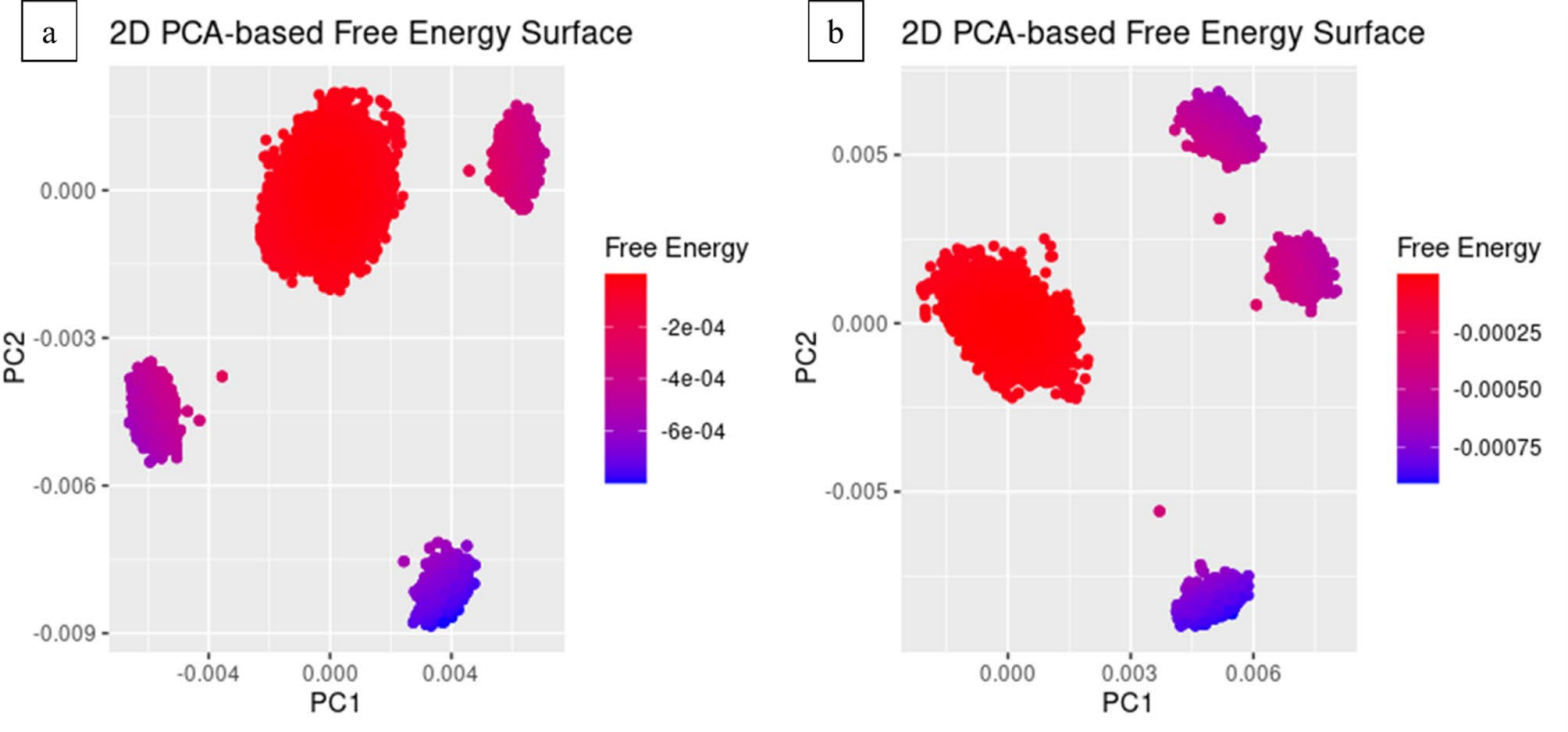
PCA-based free energy surface of the complex calculated during simulation. (**a**) CSC101378549 and (**b**) MCULE-2,532,030,345.

Principal component analysis (PCA) was performed to calculate the variance percentage within protein clusters. Moreover, it helps identify significant collective motions of complexes. The dominant movements were observed in the first five eigenvectors in both complexes. The eigenvalues in **CSC101378549** complex were 21.6, 36.1, 46.4, 52.4, and 66% in the first five eigenvectors, respectively. The plotted PCs depict < 50% of the total variations. The highest fluctuations were observed in PC1 (21.61%), while PC3 exhibited minimal variation of 10.25% compared to the others (Fig. 12a). Similarly, the eigenvalues in the first five eigenvectors of **MCULE-2,532,030,345** were 39.2%, 56.5%, 63.5%, 68%, and 77.4%, respectively. The highest variation was observed in PC1, which recorded 39.18% fluctuations during simulation. Contrastingly, 7.04% variation was observed in PC3, depicting stabilized binding (Fig. 12b). The PCA-based 2D energy surface was also generated to calculate the configurations with stable thermodynamic values. The energy surface calculated the fluctuation direction of energy in two PCs (PC1 and PC2) for carbon alpha atoms by employing the Boltzmann relation^64^. Most of the clusters lie in the local minima region depicted in purple color, indicating a stable transition from one configuration to another (Fig. 13).

### Maps of positive and negative correlation motions

**Fig. 14:**
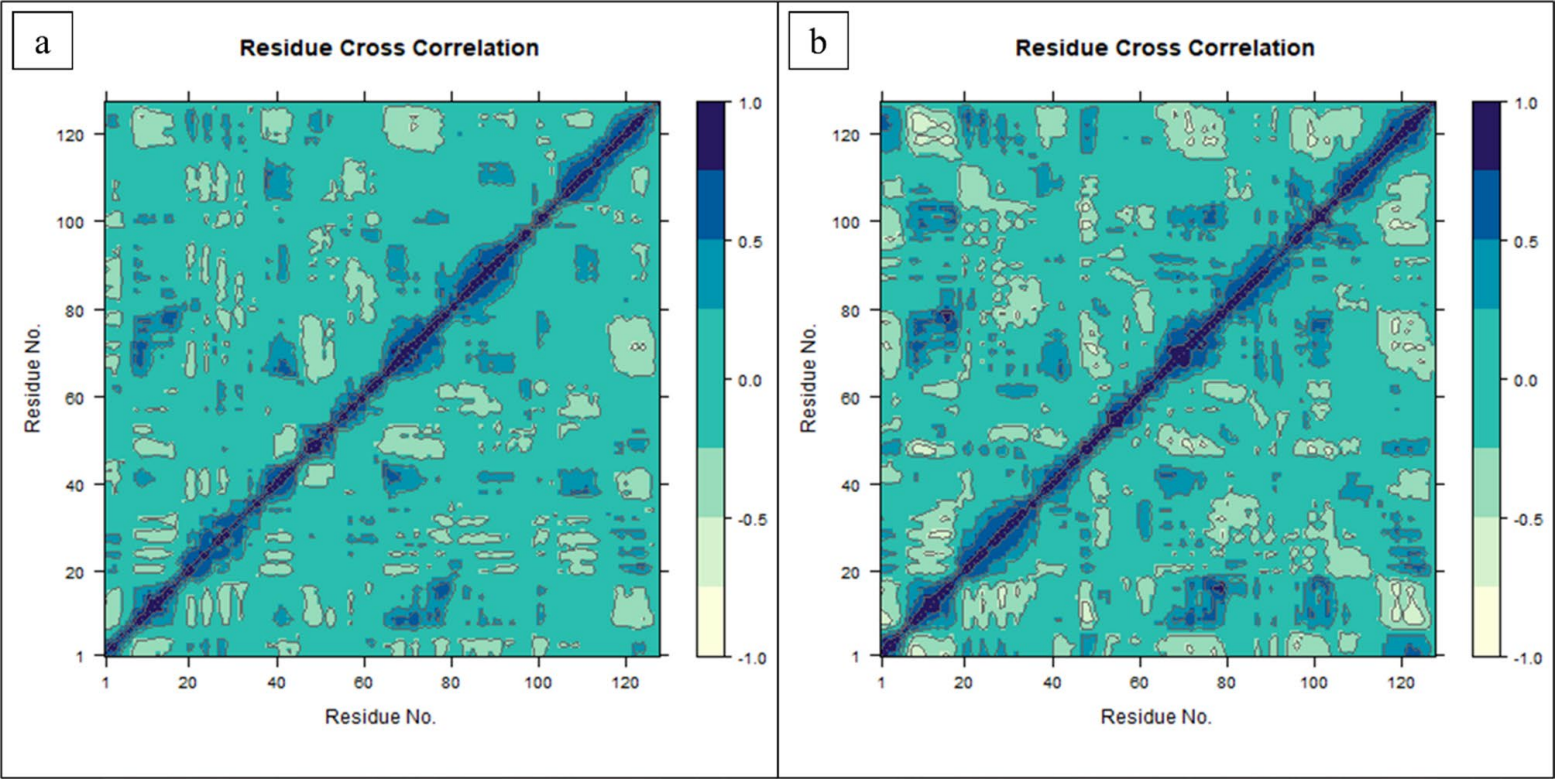
Dynamic Cross Correlation Matrix (DCCM) for (**A**) CSC101378549 and (**B**) MCULE-2,532,030,345 systems from 200ns MD simulations. The extents of correlated and anti-correlated motions are color-coded from blue to skin tone.

The dynamic cross-correlation matrix (DCCM) provides insights regarding positively and negatively correlated motions over time within both CSC101378549-BRD4 and MCULE-2,532,030,345-BRD4 systems at residual levels (Fig. 14). The blue and skin (lighter) color intensity in this heatmap represents correlated and anticorrelated motions, respectively, and assists in analyzing collective motions. According to current findings, the cutoff criteria for positively correlated residues lie above 0.8, while those <-0.4 for negatively correlated residues^65^. The blue diagonal line indicates a highly positive correlation of residues with themselves, displaying receptor rigidity too^66^. The residues range from 20 to 45, and 80 to 110 depict ligand-induced positively correlated motions. Moreover, the DCC matrix displays correlation with neighboring residues in both complexes. Resultantly, the matrix demonstrates stability in both complexes, while active site residues are involved in binding.

## Discussion

BRD4, also known as bromodomain-containing protein 4, is involved in various biological processes, including transcriptional regulation and the progression of the cell cycle. The correlation of BRD4 dysregulation with various diseases, such as neuroblastoma, has underscored its potential as a therapeutic target. The integrative pharmacoinformatic approach discussed here aims to speed up the discovery of novel compounds that can inhibit BRD4^10,67^.

Pharmacophores were used to virtually screen the five databases (Enamine, MCULE, Chemspace, ChemDiv, and CHEMBL). In order to determine the optimal binding modes, the screened hits were then docked to the BRD4 active site. Pharmacophore modeling allows for the identification of critical chemical features required for effective ligand binding. Using this method, compounds with specific pharmacophoric properties in connection to BRD4 inhibition are found by searching through large chemical databases. The virtual screening step reduces the potential inhibitor count in the pool by functioning as an initial filter^68^.

Using the chemical characteristics of a BRD4 protein co-crystal ligand (73B) (PDB ID: 4BJX), a pharmacophore model was created. The virtual screening model was developed using the four pharmacophoric properties of co-crystal ligands: hydrogen bond acceptor, hydrogen bond donor, aromatic, and hydrophobic. Based on these features, a pharmacophore-based virtual screening of five databases was carried out, and 1089 hits that satisfied the screening requirements were selected.

The PDB was used to obtain the crystal structure of the BRD4 protein. Using data from the PDB database, the crystal structure of the BRD4 protein (PDB ID: 4BJX) was retrieved and prepared for docking purposes. The hit compounds discovered during virtual screening were docked to the prepared BRD4 receptor using the Glide tool’s standard precision mode to ascertain binding affinities. This stage makes it easier to forecast whether the chemicals will be able to inhibit BRD4’s enzymatic activity^24,69^. The top ten compounds were chosen for further investigation based on their binding affinities, interactions, distances, and RMSD scores. The chosen chemicals’ binding affinities varied from − 9.623 to -8.894 kcal/mol, RMSD ≤ 1 Å, and the distance among interacting residues and ligands was ≤ 3 Å. The selected compounds’ binding affinities indicated that they would hinder the BRD4 protein’s ability to function. The known pharmaceutical agents, RVX-208 and MS436, serve as benchmarks in molecular docking and depict that selected compounds (CSC101378549 and MCULE-2532030345) display potential BRD4 inhibition capability.

We examined the molecular interactions between the selected hits and the binding pocket of the BRD4 receptor. Alkyl, carbon, conventional, van der Waal, pi-pi stacking, pi-sulfur, and pi-sigma interactions were the most often found interactions. These interactions have a significant impact on each of the top candidate compounds’ binding affinities and docking scores. Nonpolar, uncharged hydrophobic Pro82, polar uncharged Tyr97, and Asn140 residues depict significant interactions with the targeted receptor.

Drug development also requires a critical evaluation of ADMET features. The pharmacokinetic and toxicological properties of the discovered BRD4 inhibitors are assessed computationally to offer information about their possible safety and effectiveness. Drug likeness, bioavailability, and toxicity analysis were also used to display the safety profile of compounds. The predicted biological activity of CSC101378549 and MCULE-2,532,030,345 compounds demonstrates greater inhibitory potential than other studied compounds. Early in the drug discovery process, these crucial steps eliminate potentially harmful pharmacological traits, saving time and money^70^. All of the compounds’ values fell within acceptable ranges when the expected ADMET properties of the chosen compounds were examined.

Following the molecular interaction analysis, the potential binding processes of each were investigated by matching the top two chemicals (CSC101378549 and MCULE-2532030345) on the co-crystal ligand. The docked molecules showed a comparable binding mechanism and were positioned suitably on the co-crystal ligand, according to the study. This analysis supports the accuracy and reliability of the docking protocol. Additionally, it assists in confirming that hits interact with active site residues.

With Molecular Dynamics (MD) simulations, the dynamic behavior of BRD4-inhibitor complexes across time is also examined. This computational method offers useful insights into structural alterations that might impact the inhibitor’s effectiveness, allowing researchers to explore the stability and flexibility of binding interactions^71^. Flexibility and rigidity of ligands play a significant role in influencing their binding interactions with the BRD4 receptor. Flexible ligands have more rotatable bonds, which enable them to occupy several conformations and better adjust in the binding pocket of the targeted receptor. On the other hand, rigid ligands may fit more accurately into well-defined binding pockets but with more specific interactions and lack flexible binding sites too^72^. In the current study, both compounds depict moderate flexibility and rigidity (as reflected in RMSD and RMSF profiles during MD simulations) following the stable interactions with the receptor. CSC101378549 and MCULE-2,532,030,345 continued to be potent inhibitors inside the protein binding area, according to molecular dynamics simulations. Both of the compounds show stability and mobility with RMSD and RMSF values of ≤ 3 Å and 1.6 Å, respectively, strengthening the reliability of the docking results. Moreover, they depict H-bonds with Gln85, Tyr97, Asn135, Tyr139 residues, water bridges with Tyr97, Asn135, Tyr139 residues, and hydrophobic interactions with Phe83, Val87, Leu94 residues mainly. Some of the residues show more than one type of interaction, i.e., Tyr97 and Asn135; such residues play a significant inhibitory role in the active site of BRD4 receptor. On the other hand, compactness and protein folding were assessed through analyses such as radius of gyration and SASA, whose values lie within an acceptable range. Additionally, collective, correlated, and anti-correlated motions were analyzed via PCA and dynamic cross-correlation analysis^34^. According to all of these results, the selected hit compounds could function as lead compounds, which would reduce the biological activity of BRD4.

## Conclusion

This study presents an integrated pharmacoinformatic strategy for the identification of novel BRD4 inhibitors with potential application in neuroblastoma treatment. By combining pharmacophore-based virtual screening, molecular docking, ADMET prediction, and molecular dynamics simulations, we effectively narrowed down a large chemical space to a shortlist of promising candidates. Among 1089 hits initially identified, ten compounds exhibited strong binding affinity to the BRD4 active site, with docking scores ranging from − 9.623 to − 8.894 kcal/mol. ADMET profiling indicated favorable pharmacokinetic and toxicity profiles, while 100 ns molecular dynamics simulations confirmed the conformational stability of the top ligand-receptor complexes.

Beyond the identification of potential lead compounds, this study demonstrates the power of In silico workflows to accelerate early-phase drug discovery and reduce the cost and time associated with experimental screening. The findings support the continued exploration of BRD4 as a therapeutic target in oncology and highlight a rational pipeline that can be adapted for other epigenetic targets. Future experimental validation of the lead compounds will be essential to advance them toward preclinical development.

## Data Availability

Data is provided within the manuscript.
